# Estrategia farmacoinvasiva en Latinoamérica ¿ por qué no la vemos como nuestra opción?

**DOI:** 10.47487/apcyccv.v4i4.355

**Published:** 2024-03-19

**Authors:** Alexandra Arias-Mendoza

**Affiliations:** 1 Instituto Nacional de Cardiología Ignacio Chávez. Ciudad de México, México Instituto Nacional de Cardiología Ignacio Chávez Ciudad de México México

*Sr. Editor:*

La estrategia farmacoinvasiva (EF) en Latinoamérica como terapia de reperfusión en el infarto agudo de miocardio con elevación del ST (IAMCEST) es una opción viable y apegada a la realidad de nuestros países. Aunque se ha avanzado en la implementación de la estrategia farmacoinvasiva en el IAMCEST, aún existen desafíos importantes como la baja disponibilidad de salas de hemodinamia 24/7, falta de ambulancias y de redes de atención para atender oportunamente a los pacientes, especialmente en comunidades remotas y menos desarrolladas. La disparidad en la disponibilidad de servicios médicos especializados puede llevar a retrasos en el tratamiento, con consecuencias negativas para el pronóstico de los pacientes.

Es fundamental que los sistemas de salud en Latinoamérica fortalezcan sus capacidades y garanticen un acceso rápido y equitativo a procedimientos de reperfusión, ya sea a través de intervención coronaria percutánea primaria (ICP-p) o mediante el uso de estrategia farmacoinvasiva. Esto implica no solo la capacitación de los profesionales de la salud, sino también la mejora de la infraestructura para garantizar la pronta atención a los pacientes en cualquier lugar de la región.

El artículo: «Propuesta de manejo inicial del infarto de miocardio con elevación del segmento ST no complicado en centros sin capacidad de intervención coronaria percutánea en el Perú» ^1^ describió la necesidad de reperfundir de acuerdo a los recursos y logística del país, y cómo en la mayoría de los países latinoamericanos la estrategia farmacoinvasiva es la opción más viable.

Los resultados en México ^2^ con la estrategia farmacoinvasiva, donde la poca disponibilidad a salas de hemódinamica es una de las barreras para conseguir reperfusión, nos han demostrado el beneficio de esta estrategia disminuyendo desenlaces cardiovasculares. Hay que recalcar que dicha estrategia combina la fibrinolisis y la intervención coronaria temprana en la primeras 24 h; actualmente no podemos pensar en fibrinolisis como única forma de reperfusión pues el metaanálisis de Keeley ^3^ demostró la superioridad de la ICP-p, por lo que todo paciente fibrinolizado debe ser llevado a ICP para evitar el reinfarto que ocurre alrededor del tercer día, con resultados equiparables a la angioplastía primaria. Veinte años después de este metaánalisis tenemos que hablar de farmacoinvasiva, donde la fibrinolisis siempre se debe completar con ICP temprana entre las 2 a 24 h, este tiempo fue obtenido en el metaanálisis de farmacoinvasiva publicado en el 2010 ^4^, sin olvidar que algunos de los estudios incluidos como el Gracia 2, Caress AMI y TRANSFER AMI ^(^^5^ llevaban a sus pacientes a ICP en tiempos mayores a 24 h.

Desde Hace más de 10 años la estrategia farmacoinvasiva ha demostrado ser igual de efectiva que la ICP-p cuando no puede realizarse dentro del tiempo adecuado, sin olvidar que no podemos hablar solo de fibrinolisis como única terapia de reperfusión, por lo que siempre debe completarse con ICP; algunas guías hablan de referir solo a los de alto riesgo para completar la estrategia, que es una conducta inadecuada, ya que un paciente infartado y fibrinolisado es un paciente de riesgos y con alta probabilidad de reoclusión cuyo desenlace puede ser fatal.

En nuestra red de atención del valle de México, algunas veces no ha sido posible que los pacientes completen la estrategia farmacoinvasiva en las primeras 24 h y un 15% de los fibrinolisados completan la estrategia en 48 h. Nuestra última publicación ^(^^6^^)^ demostró que cuando se esperan retrasos en los pacientes y en el sistema, la estrategia farmacoinvasiva se asoció con una duración de estadía y mortalidad similares, sin mayores costos a las ICP-p y con eventos cardiovasculares más bajos y, además, la estrategia es rentable en comparación con pacientes no reperfundidos.

Las guías europeas ^(^^7^ del 2023 sugieren que los pacientes fibrinolizados deberían someterse a una ICP dentro de las 2 - 24 h posteriores a la fibrinólisis. El tiempo óptimo para la ICP después de la fibrinólisis puede variar según la estabilidad hemodinámica del paciente, la disponibilidad de recursos y la experiencia del centro médico. En algunos casos la ICP puede ser realizada tan pronto como sea posible después de la fibrinólisis, especialmente si hay signos de reoclusión o inestabilidad clínica.

En resumen, insto a la comunidad médica y a las autoridades de salud en Latinoamérica a realizar esfuerzos para garantizar un tratamiento farmacoinvasivo oportuno y efectivo en el IAMCEST. La inversión en la mejora de la infraestructura, la concientización, la educación pública sobre los síntomas de un infarto y la importancia de buscar atención médica de manera inmediata, la capacitación continua del personal médico y la creación de redes son pasos esenciales para mejorar los desenlaces cardiovasculares y disminuir los tiempos de atención. Mejorar la terapia de reperfusión en el infarto en Latinoamérica es un objetivo crucial para optimizar los pronósticos y la calidad de vida de los pacientes y así contribuir al fortalecimiento de las estrategias de atención a pacientes con infarto en nuestra región.
